# Exploring Gender-Sensitive Serious Games for Nutrition Communication: A Formative Qualitative Study in Rural Indonesia

**DOI:** 10.3390/ijerph23030390

**Published:** 2026-03-18

**Authors:** Netty Dyah Kurniasari, Iriani Ismail, Prita Dellia, Ana Tsalitsatun Ni`mah, Iswari Hariastuti

**Affiliations:** 1 Department of Communication Science, Faculty of Social and Cultural Sciences, Universitas Trunodjoyo Madura, Bangkalan 69162, Indonesia; 2 Department of Management, Faculty of Economics and Business, Universitas Trunodjoyo Madura, Bangkalan 69162, Indonesia; iriani.ismail@trunojoyo.ac.id; 3 Department of Informatics Education, Faculty of Teacher Training and Education, Universitas Trunodjoyo Madura, Bangkalan 69162, Indonesia; prita.dellia@trunojoyo.ac.id (P.D.); ana.tsalits@trunojoyo.ac.id (A.T.N.); 4 Research Center for Public Health and Nutrition, National Research and Innovation Agency (BRIN), Jakarta 10340, Indonesia; iswarihariastuti@yahoo.com

**Keywords:** gender-sensitive serious games for nutrition communication, stunting, rural Indonesia, trust-based communication, AI-supported features, formative qualitative study

## Abstract

**Highlights:**

**Public health relevance—How does this work relate to a public health issue?**

- This study examines the ongoing issue of childhood stunting in rural Indonesia, where gender norms and inadequate nutritional knowledge impede effective solutions.
- It investigates culturally rooted communication tactics via serious games for moms, young women, and families residing in high-stunting areas.

**Public health significance—Why is this work of significance to public health?**

- The research illustrates that gender-sensitive, game-based interventions might enhance nutritional knowledge and decision-making in high-risk populations, such as moms and brides-to-be.
- Findings emphasise the necessity of incorporating local culture, religion, and family dynamics to improve the acceptance of digital nutrition teaching tools.

**Public health implications—What are the key implications or messages for practitioners, policy makers and/or researchers in public health?**

- Gender-sensitive serious games can enhance national stunting-reduction initiatives by providing interactive, accessible, and engaging educational experiences.
- The findings offer practical insights for public health professionals, politicians, and Puskesmas.

**Abstract:**

Stunting remains a major public health challenge in Indonesia, with a national prevalence of 21.6% in 2022. Rural regions such as Madura face heightened vulnerability due to cultural dietary taboos, gendered caregiving structures, intergenerational authority, and digital disparities that shape household nutrition decision-making. This formative qualitative study explores stakeholders’ perceptions to inform the conceptual development of gender-sensitive serious games for nutrition communication in rural Indonesia. Using an exploratory design, 42 informants, including mothers of children under five, brides-to-be, health cadres, midwives, religious and community leaders, and local digital actors, were recruited across rural Madura. Thematic analysis examined trust-based communication patterns, gender dynamics, perceptions of artificial intelligence (AI), and contextual conditions influencing digital health acceptance. Findings indicate that acceptance of gender-sensitive serious games depends on cultural alignment, institutional endorsement, perceived credibility, and usability in low-resource settings. Participants consistently positioned serious games and AI-supported features as complementary communication layers rather than replacements for health workers. Game-based tools were considered potentially relevant when designed to support intergenerational co-play, integrate local narratives and religious values, and function in low-connectivity environments. Rather than evaluating an implemented intervention, this study proposes a conceptual design framework grounded in feminist communication perspectives, serious games scholarship, and technology acceptance theory. The findings provide context-sensitive insights to guide future prototype development and pilot testing within hybrid, community-based nutrition communication systems.

## 1. Introduction

Malnutrition and inadequate nutrition literacy remain major global public health challenges, particularly among women and children in low- and middle-income countries (LMICs). Despite sustained international efforts aligned with the Sustainable Development Goals (SDGs), undernutrition continues to contribute significantly to maternal and child morbidity and long-term developmental risks. Beyond biomedical interventions, effective health communication is increasingly recognised as a critical determinant of nutrition-related knowledge, attitudes, and household practices.

In recent years, digital health technologies, including artificial intelligence (AI), have been explored as potential tools to enhance health communication through personalisation, interactivity, and broader reach. AI-supported chatbots, gamified applications, and digital nudging strategies have demonstrated potential to facilitate engagement and food literacy across diverse populations. However, this study specifically focuses on the conceptualisation of gender-sensitive serious games for nutrition communication, rather than AI-based clinical systems. In rural and underserved settings, AI-enabled systems have also been applied in telemedicine, clinical decision support, and health monitoring to address workforce shortages and geographic barriers [1,2]. However, evidence consistently highlights infrastructural limitations, digital divides, governance concerns, and the need for human mediation and contextual adaptation in rural environments [3,4]. These constraints suggest that technological feasibility alone does not guarantee social acceptance or meaningful integration into everyday practices.

However, the expansion of digital health technologies in LMICs is also shaped by persistent gender digital divides. Women in many LMIC contexts are less likely to access mobile devices, internet services, and digital platforms due to socio-economic inequalities, educational disparities, and restrictive cultural norms [5,6,7]. Evidence further suggests that improvements in digital infrastructure alone are insufficient without gender-sensitive digital literacy initiatives and supportive social policies [8,9]. These structural inequalities are particularly relevant in rural settings, where household hierarchies and intergenerational authority may constrain women’s access to and autonomy in digital spaces.

Within this broader digital landscape, serious games in health communication contexts are communication tools that integrate learning and entertainment. Serious games have been explored as approaches to enhance dietary awareness, physical activity knowledge, and health literacy, particularly among children and adolescents [10]. By transforming abstract nutritional guidelines into interactive experiences, serious games may foster motivation and reinforce learning through participatory engagement.

Evidence from LMIC contexts further suggests that culturally adapted serious games can support maternal and community health education when collaboratively designed with local stakeholders and adapted to local literacy needs [11,12]. For example, the MANTRA project in rural Nepal demonstrated significant improvements in maternal health knowledge among low-literacy women through co-created mobile game modules. Similar exploratory work in rural Ethiopia has indicated the potential of game-based approaches for maternal healthcare and nutrition education.

However, global evidence also highlights persistent challenges related to trust, contextual relevance, digital access, and inclusivity when introducing AI-supported or technology-driven interventions in rural or culturally conservative areas. These challenges underscore the importance of culturally grounded, gender-sensitive design approaches in digital nutrition communication, particularly in settings where health decision-making is embedded in relational and intergenerational dynamics.

In the Asian and Southeast Asian (SEA) context, nutrition education is deeply embedded within family structures, cultural norms, and community-based health practices. Food-related decision-making is often collective, shaped by intergenerational authority, religious values, and socially negotiated trust. While digital health interventions in the region have shown promise, their effectiveness depends heavily on cultural adaptation and alignment with local communication practices. Evidence suggests that participatory and family-centred learning approaches are more effective than purely individual-focused interventions, particularly in maternal and child nutrition contexts. Gamified tools incorporating storytelling, collective play, and culturally familiar narratives have demonstrated potential to enhance engagement and learning outcomes [13,14]. However, many digital nutrition initiatives remain technologically driven and insufficiently attentive to gendered power relations and culturally embedded trust dynamics.

Stunting remains a critical public health issue in Indonesia and continues to challenge national nutrition and development targets. In 2022, the national prevalence of stunting was 21.6%, remaining above the World Health Organisation (WHO) threshold [15]. In rural regions such as Madura, cultural food taboos, patriarchal gender norms, early marriage, and persistent digital divides intensify risk factors. Mothers and young brides carry primary responsibility for child feeding. However, their autonomy is often constrained by limited access to resources and reliance on elders or male household members for decision-making. Similar structural constraints have been documented in other resource-constrained settings [16].

Despite national nutrition programmes, communication strategies in rural Indonesia frequently remain top-down and insufficiently participatory. Although previous studies have examined serious games, digital nutrition education, and AI-based health communication, two key gaps remain. First, there is limited research on gender-sensitive design for AI-supported nutrition communication in rural and culturally conservative settings, particularly where gender, culture, and household power relations intersect. Second, existing digital approaches rarely integrate traditional trust-based communication networks such as health cadres, midwives, and religious leaders with emerging technologies. Community health mediation literature emphasises the importance of trusted local intermediaries in facilitating culturally grounded and relational health communication in underserved contexts [17,18].

Moreover, children are often positioned as passive recipients rather than active co-participants in family-based learning processes. Empirical exploration of children’s roles as co-players and co-learners within digital nutrition communication remains limited.

Addressing these gaps is essential for developing communication strategies that are not only technologically innovative but also socially legitimate, culturally grounded, and relationally embedded within rural communities.

This study responds to the limited empirical evidence on how gender, trust, and cultural authority intersect in shaping the acceptance of AI-supported nutrition communication in rural Southeast Asian contexts.

### Research Objectives

Therefore, this study aims to examine how gender-sensitive serious games function as participatory tools for nutrition communication among mothers, children, and brides-to-be in rural Indonesia. Specifically, this study seeks to:

(1) examine stakeholders’ perceptions of the cultural acceptability and usability of gender-sensitive serious games for nutrition communication;
(2) analyse how serious games facilitate family-based nutrition learning and position children as co-players and learners; and
(3) Identify key design principles within gender-sensitive serious games for nutrition communication, including AI-supported features where appropriate

## 2. Methods

**Figure 1:**
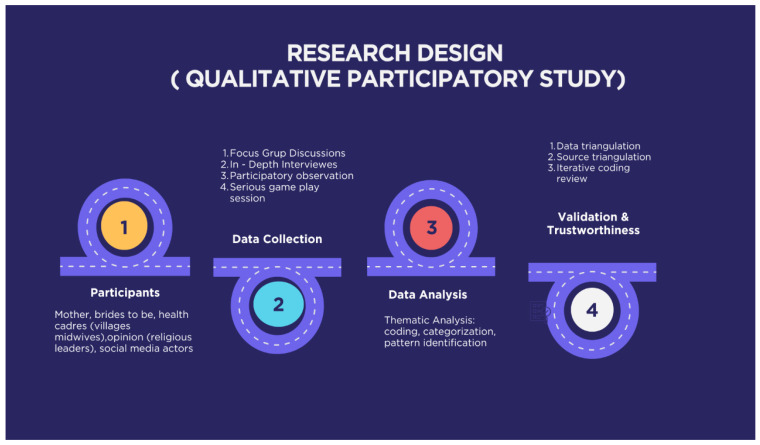
The diagram summarises the overall study design, participant groups, data collection techniques, analytical procedures, and validation strategies, enabling readers to understand the research flow before engaging with the detailed methodological description.

This study employed a qualitative, exploratory, and participatory research design to examine gendered, cultural, and communication dimensions of nutrition-related practices in rural Madura, Indonesia. The study was positioned as a pre-intervention, design-oriented inquiry, aiming to generate contextual and empirically grounded insights to inform the future development of gender-sensitive communication tools for nutrition education, rather than to evaluate intervention outcomes or measure behavioural change. To enhance methodological clarity and address the complexity of the research process, this study provides a visual overview of the methodology (Figure 1).

**Figure 2:**
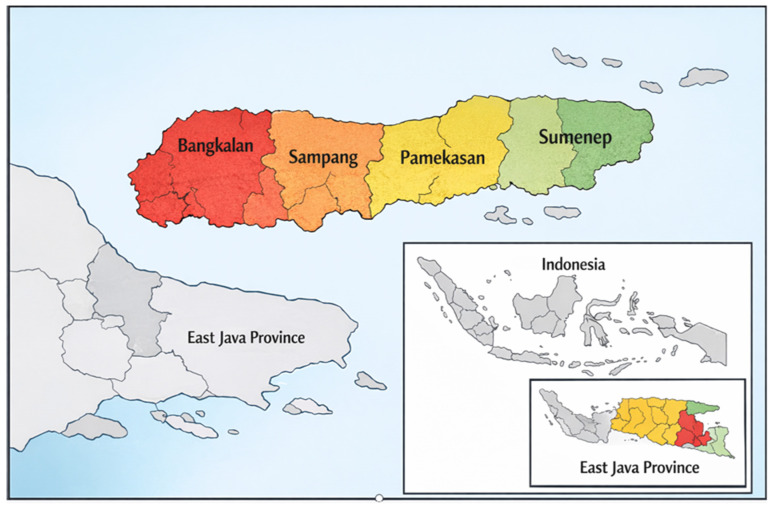
Map of Madura Island, East Java Province, Indonesia, showing the study area. The research was conducted in three districts: Bangkalan, Pamekasan, and Sumenep, which represent rural communities within the Madurese socio-cultural context. Source: Authors’ adaptation.

The study involved 42 participants representing key community roles related to household nutrition practices and health communication. Participants were purposively selected to ensure diversity across gender, age, and social authority roles. The sample included mothers of children under five, brides-to-be and young women, health cadres, village midwives, community and religious leaders, and local social media actors drawn from rural communities across Madura Island, East Java (Figure 2). The characteristics of the participants are presented in Table 1.

Of the 42 participants, the majority were women (*n* = 30), reflecting their central roles in household nutrition practices. Male participants (*n* = 12) included community leaders, social media actors, and one health cadre. The inclusion of male participants allowed exploration of household authority, religious influence, and community endorsement dynamics.

The inclusion of diverse participant groups enabled the triangulation of perspectives across household, community, and institutional levels. Mothers and brides highlighted personal challenges related to food taboos and decision-making authority, while cadres and midwives contributed professional insights into nutrition guidance. Community and religious leaders emphasised the role of cultural norms and trust in shaping nutrition-related behaviours.

The semi-structured interview and discussion guides were developed to elicit participants’ experiences, perceptions, and social contexts regarding nutrition practices and health communication. Core guiding questions included:

1. Household nutrition practices

Can you describe daily feeding practices for mothers and children in your household or community?

2. Cultural and dietary norms

Are there any cultural, traditional, or religious beliefs that influence food choices for mothers, children, or pregnant women?

3. Gendered decision-making

Who usually makes decisions about food preparation and child nutrition in the household, and why?

Access to and trust in nutrition information

*What sources of nutrition or health information do you trust the most, and what makes these sources credible for you?*

4. Role of community health actors

How do health cadres, midwives, or community leaders support nutrition education in this community?

5. Perceptions of digital and AI-based tools

What are your views on using digital tools, mobile applications, or artificial intelligence to support nutrition education?

6. Acceptability of serious games

How do you feel about the idea of using games or interactive digital activities to learn about nutrition for mothers, children, or couples preparing for parenthood?

7. Design preferences and contextual adaptation

What features would make a digital nutrition game acceptable and useful in your community?

Data collection methods included semi-structured in-depth interviews, focus group discussions, participatory observation of community health activities (including Posyandu), and facilitated interaction sessions with a serious game. These complementary methods enabled the triangulation of perspectives across household, community, and institutional levels.

All qualitative data were analysed using inductive thematic analysis. First, interview transcripts and field notes were repeatedly read to achieve data familiarisation. Second, initial codes were generated to capture meaningful units related to nutrition practices, gender relations, and communication experiences. Third, codes were compared and clustered into preliminary themes. Fourth, themes were reviewed and refined through iterative discussion among the research team. Finally, the themes were interpreted in relation to feminist communication theory, serious games literature, and perspectives on technology acceptance.

### Data Validation and Trustworthiness

To ensure data validity and trustworthiness, this study employed triangulation across data sources and data collection methods, including in-depth interviews, focus group discussions, participant observations, and serious game interaction sessions. Data analysis was conducted through an iterative thematic coding process, with regular cross-checking and discussion among the research team to enhance analytical consistency. These procedures contribute to the credibility, dependability, and rigour of the qualitative findings. Data collection continued until thematic saturation was reached across participant groups. The sample size was considered adequate for exploratory qualitative inquiry, as thematic repetition was observed across participant groups and no substantially new categories emerged in later interviews. Coding was conducted independently by two researchers and discussed to reach analytical consensus. Reflexive discussions were held throughout the analytical process to minimise interpretative bias and enhance contextual sensitivity.

Particular attention was given to participants’ perceptions and expectations regarding the potential role of gender-sensitive serious games as communication tools for nutrition education, particularly with respect to cultural relevance, usability, and community acceptance. These insights informed the development of a conceptual design framework rather than serving as evidence of implemented or evaluated interventions.

## 3. Results

### 3.1. Contextual Background

The findings are situated in rural Madura, where nutrition communication unfolds within gendered caregiving structures, strong intergenerational authority, and community-based health systems such as Posyandu. Although national nutrition programmes and health services are present, everyday nutrition practices are shaped by cultural food taboos, household hierarchies, and varying levels of digital access. Participants’ accounts reflect how mothers, brides-to-be, children, and community actors negotiate nutrition-related information within these structural and social conditions. The following sections present themes derived from participants’ experiences.

#### 3.1.1. Trust-Based Nutrition Communication in Rural Madura

Participants consistently described child stunting in rural Madura as shaped by interconnected social, cultural, and gendered factors that influence how nutrition information is accessed, trusted, and applied in everyday household contexts. Across interviews with mothers, brides-to-be, healthcare workers, village midwives, and religious leaders, nutrition-related decision-making was rarely based solely on information but rather was negotiated through family hierarchies, cultural norms, and trust in specific sources of authority.

#### 3.1.2. Mothers as Primary Caregivers Within Gendered Decision-Making Structures

Across study sites, mothers were consistently identified as the primary actors responsible for daily child feeding and nutrition-related practices. They actively accessed information through midwives, Posyandu cadres, social media, and peer networks, positioning themselves as central managers of household nutrition.

“I usually obtain information about child nutrition from midwives, Posyandu cadres, and mothers’ WhatsApp groups.”

(Mother A, 24)

“I usually get nutrition information from social media and from Posyandu.”

(Mother SK, 29)

“I usually get nutrition information from the village midwife during immunisation sessions and from mothers’ WhatsApp groups. I also ask neighbours when my child has difficulty eating.”

(Mother, 31, Sumenep)

These accounts illustrate that mothers do not passively receive information but actively navigate multiple interpersonal and digital sources.

Mothers with prior health education backgrounds expressed greater confidence in interpreting and applying nutrition guidance.

“I studied in the health field, so most of my nutrition information comes from my education, workplace, and online sources.”

(Mother of under-five children, 40 years, Sumenep)

However, responsibility did not automatically translate into decision-making autonomy. Nutrition practices were often negotiated within gendered household structures, particularly in extended families where elders and spouses influenced final decisions.

“Sometimes people say eggs cause fever, but the midwife explained that this is only a myth. I feel confused when the advice from family members differs from that of health workers.”

(Mother ER, 25)

Similarly, another mother reflected on the relational nature of nutrition decisions:

“Mothers of young children described themselves as the primary actors responsible for daily feeding practices and child nutrition. However, their decisions were not made in isolation. Nutrition-related choices were often negotiated within family structures, shaped by elders’ advice and socially accepted norms. While mothers actively received information from health services, applying this knowledge consistently at home required navigating household expectations and economic constraints.”

(Ummi, mother of an under-five child, 29 years, Bangkalan)

Across accounts, mothers emerged as key negotiators between biomedical advice, digital information, and familial expectations. Their caregiving role was central, yet embedded within relational and gendered decision-making structures that shaped how nutrition knowledge was interpreted and enacted. (Informants: Mothers of under-five children, ages 24–40, Bangkalan and Sumenep)

#### 3.1.3. Male Perspectives on Authority and Support

Although mothers were central to daily caregiving, male participants, including community leaders, religious figures, and young men preparing for marriage, emphasised their roles in endorsing, legitimising, or mediating nutrition practices within households. Male informants frequently framed their involvement in terms of moral authority, religious guidance, or structural support rather than in terms of direct caregiving. This distinction highlights how gendered authority operates differently from gendered responsibility in rural nutrition communication.

Several male participants positioned themselves as figures who reinforce or validate nutrition messages within broader moral and community frameworks. For instance, a religious leader explained:

“Information delivered by religious leaders is the most trusted.”

(Religious leader, 52 years, Sumenep)

Similarly, a community leader emphasised the importance of collective endorsement:

“If information comes from community leaders or religious figures, people are more likely to listen and follow it.”

(Community leader, 52 years, Pamekasan)

A young man preparing for marriage reflected limited direct involvement in caregiving responsibilities, positioning himself more as a learner than a decision-maker:

“I do not really understand nutrition yet. Usually, I follow advice from my parents or older family members.”

(Bride-to-be [young man], 25 years, Sampang)

These accounts indicate that while daily nutrition management was largely associated with women, men were described as influential actors within household and community authority structures. Their roles were framed less in terms of routine caregiving and more in relation to moral validation, social endorsement, and institutional trust.

#### 3.1.4. Dietary Taboos and Conflicting Sources of Authority

Participants reported the persistence of dietary taboos related to pregnancy, breastfeeding, and early childhood feeding. These beliefs often conflicted with biomedical recommendations, creating uncertainty in daily decision-making.

“People say that if a child eats eggs, it will cause internal heat. The midwife said it is not true, but family members still forbid it.”

(Mother, 25, Pamekasan)

“Sometimes nutrition practices clash with family beliefs, such as myths that prohibit mothers from eating certain nutritious foods after childbirth.”

(Mother of under-five children, 40 years, Sumenep)

Such accounts reflect how nutrition guidance is negotiated within intergenerational authority structures. Advice from elders frequently carried strong normative weight, even when it contradicted professional health recommendations.

Conflicting messages were also amplified through digital platforms. Participants described confusion when encountering inconsistent advice on social media.

“Sometimes I feel confused because there are many nutrition myths on social media.”

(Bride-to-be, 19 years, Sumenep)

“I once experienced confusion when a mother was not allowed to eat protein after giving birth because it was believed it could affect the baby’s nutrition.”

(Local social media actor, 26 years old, Sumenep)

While digital media expanded access to information, it also intensified exposure to misinformation, requiring mothers and young women to navigate multiple, sometimes competing, sources of authority.

Religious leaders further emphasised that nutrition information was often evaluated through moral and religious lenses.

“Many nutrition messages on social media are not in line with religious values, so people here trust information delivered by religious figures more.”

(Religious leader, 52 years, Sumenep)

In such contexts, religious figures accompanying youth activities were perceived as important reference points to validate nutrition messages. When uncertainty arose, mothers and young women frequently relied on trusted social or religious authorities rather than solely on digital sources.

Overall, dietary taboos and conflicting authority structures created a layered communication environment in which biomedical advice, family traditions, digital narratives, and religious legitimacy intersected. These dynamics illustrate that nutrition communication in rural Madura operates within negotiated social hierarchies rather than within a linear information-transfer model. (Informants: Mothers of under-five children, brides-to-be, religious leaders, and local media actors, ages 19–52, Bangkalan, Pamekasan, and Sumenep).

#### 3.1.5. Early Marriage and Limited Nutrition Preparedness Among Young Women

Young women preparing for marriage actively sought nutrition information, primarily through digital platforms and pre-marital programmes, illustrating early awareness but limited experiential preparedness.

“I get nutrition information from pre-marital classes at the health centre and from the District Health Office’s Instagram. I want to be ready to become a mother later.”

(Bride-to-be, 19 years, Sumenep)

Despite proactive information-seeking, participants acknowledged that their understanding remained fragmented and highly dependent on mediated sources rather than hands-on practice.

Young women and brides-to-be described limited preparedness for nutrition-related responsibilities, often relying on older family members for guidance. Emphasising their potential to make independent decisions can help the audience see opportunities for empowerment.

“I do not really understand nutrition yet. Usually, I follow advice from my parents or older family members.”

(Bride-to-be, 20 years)

Early marriage shaped nutrition communication, with brides-to-be and young mothers heavily dependent on senior family members, especially mothers-in-law, which often diminished their confidence in applying formal health advice.

As one bride-to-be stated:

“I usually follow my mother-in-law’s advice about nutrition during pregnancy. Sometimes I feel it is unfair, but I am hesitant to disagree”

(Bride AN, 19 years).

These narratives highlight intergenerational power relations that shape how nutrition information is interpreted and enacted.

Young adults preparing for marriage demonstrated openness to learning about child nutrition and digital health tools, but also expressed uncertainty about their accuracy and relevance. Recognising this can motivate the audience to support accessible, trustworthy information sources. (Informant 2—Bride-to-be (young man), 25 years, Sampang)

Young individuals preparing for marriage described limited prior exposure to structured nutrition education. Nutrition information was often perceived as something to be learned later, after marriage or pregnancy. This resulted in low preparedness regarding maternal and child nutrition, despite awareness of stunting as a public health issue. (Usman Yunus, bride-to-be, 19 years, Bangkalan)

Young women, particularly brides-to-be and recently married adolescents, demonstrated limited preparedness for nutrition-related responsibilities. Their understanding of maternal and child nutrition was often fragmented and heavily reliant on pre-marital classes, social media, or advice from older female relatives. While younger informants expressed openness to learning from and using digital sources, they also reported uncertainty about evaluating the credibility of information. Early marriage thus intersected with gaps in experiential knowledge, increasing dependence on informal guidance during pregnancy and early childcare. (Informants: Brides-to-be and young women, ages 19–23, Bangkalan and Sumenep)

#### 3.1.6. Gaps Between Formal Health Education and Daily Household Practices

Although formal nutrition education was widely accessible through Posyandu sessions, puskesmas services, and community-based campaigns, participants described persistent gaps between knowledge acquisition and everyday household practice. Health communication was generally perceived as informative; however, sustaining recommended behaviours in routine settings remained challenging.

“During Posyandu sessions, healthy food is explained, but at home it is sometimes difficult to apply because of long-standing habits.”

(Mother A, 24)

“Sometimes the information I receive is different and confusing.”

(Mother SK, 29)

These accounts suggest that exposure to information does not automatically translate into consistent practice. Habitual routines, family expectations, and economic constraints often shaped how nutrition guidance was interpreted and implemented.

From an institutional perspective, formal nutrition information was described as standardised and evidence-based. However, confusion persisted at the community level due to misinformation and competing narratives.

“Information that uses survey data is more trustworthy, but there are still many hoaxes circulating on social media.”

(Public health officer, 34 years, Sumenep)

“Information that uses national data is more trusted, but many people are still confused because of hoaxes.”

(Health communication officer, 30 years, Sumenep)

At the household level, application of nutrition guidance was shaped not only by clarity of information but also by structural conditions.

“Nutrition information is much better now than in the past because access is easier, but not everyone can apply it due to different social and economic conditions.”

(Mother of under-five children, 40 years, Sumenep)

Health cadres further emphasised that reinforcement and mediation were often necessary for sustained engagement.

“Information from social media is sometimes confusing. Mothers trust information more when it has an official government label.”

(Health cadre, 42 years, Sumenep)

“We explain balanced nutrition, but after the meeting, many people return to their habits. They often need reminders.”

(Cadre PS, 40)

These narratives indicate that formal information delivery—while essential—does not function independently of social mediation. Participants also noted limited proactive information-seeking behaviour among some community members.

“I rarely search for nutrition information myself. Usually, information comes from health officers or pamphlets.”

(Religious leader, 52 years, Sumenep)

Similarly, brides-to-be described exposure to digital content without sustained engagement in structured education.

“I have never joined a direct nutrition campaign. Most of the information I see is on social media.”

(Bride-to-be, 19 years, Sumenep)

Together, these findings illustrate a recurring gap between institutional knowledge production and everyday household practice. Rather than reflecting a lack of awareness, this gap appears shaped by structural constraints, habitual patterns, social negotiation, and fragmented digital information environments. (Informants: Mothers, brides-to-be, health cadres, health officers, and religious leaders, ages 19–53, Bangkalan, Sumenep, and Pamekasan)

### 3.2. Roles of Community and Institutional Actors in Nutrition Communication

Participants emphasised that nutrition communication in rural Madura is strongly shaped by the roles of community and institutional actors who mediate information, establish trust, and influence everyday practices. Beyond household-level decision-making, the circulation and acceptance of nutrition messages were closely linked to the presence of trusted intermediaries, including health cadres, village midwives, religious leaders, and local digital actors.

Although health education was delivered through formal channels such as Posyandus and health services, translating this information into daily routines remained challenging. Practical constraints, habitual practices, and family influence contributed to gaps between knowledge and action at the household level. (Ummi, mother of an under-five child, 29 years, Bangkalan; Nurhayati, Posyandu cadre, 45 years, Pamekasan).

#### 3.2.1. Health Cadres and Village Midwives as Trusted Intermediaries

Health cadres and village midwives are recognised as highly reliable sources of nutrition information at the community level. Their credibility, built through routine interaction, familiarity, and ongoing engagement with families, fosters trust and confidence in their guidance.

“People trust information more when it comes from someone they know directly, such as midwives or Posyandu cadres.”

(Female community figure, 26 years old, Sumenep)

From an institutional perspective, cadres and midwives serve as vital community health advocates, acting as extensions of public health authority at the village level.

“Midwives and health cadres are very important as the extension of the Health Office.”

(Health communication officer, 30 years, Sumenep)

Mothers similarly emphasised their reliance on these actors when seeking clarification about nutrition.

“The information I trust the most is from the midwife. Village leaders and the midwife really help in providing nutrition information.”

(Mother, 31, Sumenep)

“Information from doctors, midwives, or the government is the most trusted and most frequently shared.”

(Mother A, 24)

“Doctors are the most trusted when delivering health information.”

(Mother SK, 29)

Health cadres described their role as translators of formal guidelines into locally understandable messages.

“I get information from cadre training and the Maternal and Child Health book, and then share it with mothers during Posyandu meetings.”

(Health cadre, 42 years, Sumenep)

“I usually obtain child nutrition information from routine trainings held by the health centre or district health office, from pocket books or modules provided, and from WhatsApp groups among cadres where we share information.”

(Health cadre, 31, Pamekasan)

Cadres also emphasised the relational basis of trust.

“People trust us because we come regularly and speak in ways they understand. If we explain too formally, they will forget or ignore it.”

(Cadre, 38)

Village midwives underscored that their credibility relies on professional training and evidence-based guidance, reinforcing their authority in nutrition communication.

“As a midwife, I obtain child nutrition information mainly from official trainings and seminars held by the Ministry of Health, the District Health Office, or professional organisations such as the Indonesian Midwives Association. I also regularly consult scientific journals and clinical practice guidelines.”

(Midwife, 40, Pamekasan)

Together, these accounts illustrate how cadres and midwives function as trusted intermediaries who bridge institutional health systems and everyday household practices. Their authority is derived not only from biomedical knowledge but also from sustained interpersonal relationships, social proximity, and culturally adaptive communication. (Informants: Posyandu cadres and village midwives, ages 31–45, Pamekasan and Sumenep; Mothers of under-five children, ages 24–40, Sumenep

#### 3.2.2. Religious and Community Leaders as Normative Influencers

Religious and community leaders were consistently described as influential figures in shaping community acceptance of nutrition messages. Their authority was grounded in moral credibility, social proximity, and frequent interaction within everyday village life.

“The role of community leaders, midwives, and Posyandu cadres is very important. They are the most trusted figures in the village. If information comes from them, people are more likely to listen and follow it.”

(Community leader, 52, Pamekasan)

Religious leaders were also identified as important actors in supporting youth understanding of nutrition.

“Religious leaders who accompany young people really help us understand nutrition better.”

(Bride-to-be, 19, Sumenep)

From the perspective of religious authority itself, trust was framed as deeply embedded in community structures.

“Information delivered by religious leaders is the most trusted.”

(Religious leader, 52 years, Sumenep)

Health professionals likewise acknowledged the normative role of religious endorsement in strengthening public acceptance.

“Religious leaders are the most trusted figures in the community.”

(Public health officer, 34 years, Sumenep)

Community health actors emphasised that collaboration between health workers and religious figures enhanced the legitimacy of their work.

“Health workers are the most trusted, but their messages are stronger when supported by religious leaders.”

(Mother of under-five children, 40 years, Sumenep)

Religious endorsement was particularly important when addressing dietary misconceptions and culturally rooted beliefs.

“If the religious leader supports the message, families are more willing to listen.”

(Cadre, 42 years, Sumenep)

Taken together, these accounts indicate that nutrition communication in rural Madura operates within moral and cultural frameworks in which religious and community leaders serve as key normative mediators. Their involvement reinforces legitimacy, reduces resistance, and situates health messages within culturally meaningful contexts. (Informants: Religious leaders, community leaders, mothers, cadres, and public health officers, ages 19–52, Pamekasan and Sumenep)

#### 3.2.3. Informal Digital Actors and Local Social Media Channels

Participants described the growing role of informal digital actors and local social media channels in the circulation of nutrition-related information. Platforms such as WhatsApp, Instagram, TikTok, and Facebook were frequently used to access and redistribute content within village communities.

“I get information about child nutrition through social media such as TikTok, Instagram, Facebook, and also from family and parents.”

(Local social media actor, 26 years old, Sumenep)

“I have shared nutrition information both verbally and through social media.”

(Local social media actor, 26 years old, Sumenep)

Among mothers, messaging groups functioned as everyday spaces for information exchange.

“I often share nutrition information with my family through WhatsApp.”

(Mother, 31, Sumenep)

“Yes, I have shared nutrition information, usually through family or friends’ WhatsApp groups.”

(Mother A, 24)

“Most of the information I receive comes from social media.”

(Mother SK, 29)

Community-based WhatsApp groups were also described as semi-formal spaces in which information circulated beyond face-to-face interactions.

“I usually get nutrition information from the mothers’ WhatsApp group in the village. Sometimes information is shared there, and I also pass it on to neighbours during casual conversations.”

(Community leader, 52, Pamekasan)

For young women, visual and peer-oriented platforms were particularly influential.

“I follow nutrition accounts on Instagram and often share information with friends through Instagram stories.”

(Bride-to-be, 19 years, Sumenep)

Health cadres similarly relied on digital messaging platforms for rapid coordination and exchange of updates.

“The platform we use most frequently is the WhatsApp group among cadres and health workers, because information arrives quickly and we can discuss it directly.”

(Health cadre, 31, Pamekasan)

From an institutional perspective, formal health communicators acknowledged the complementary role of local digital environments.

“We share information through direct socialisation to health centres and printed media, but local social media also plays a role.”

(Health communication officer, 30 years, Sumenep)

Local health communication increasingly incorporates social media management at the district level. (Local health communication increasingly involves digital platforms managed by institutional actors. Social media was used to disseminate simplified and visual nutrition messages, expanding reach beyond formal health settings.) (Junaidi, health social media manager, 30 years, Dinas Kesehatan Sumenep)

Despite their reach and speed, participants held mixed views on digital credibility. Trust in content depended heavily on familiarity with the source and on visible links to recognised health authorities. Information shared by known community members or associated with cadres, midwives, or official institutions was perceived as more reliable than content encountered from unfamiliar online sources.

Local social media administrators and informal digital actors were thus described as emerging communication nodes that amplify health messages beyond face-to-face settings. However, participants described that collaboration with health professionals remained important for reducing misinformation and maintaining legitimacy. (Informants: Social media managers and digital communicators, ages 26–30, Sumenep; mothers and community leaders, Sumenep and Pamekasan)

#### 3.2.4. Hybrid Communication Pathways (Offline–Online)

Participants consistently described nutrition communication as operating through hybrid pathways that combine face-to-face interaction with digital dissemination. Rather than replacing offline engagement, digital platforms were perceived as reinforcing and extending messages delivered through trusted interpersonal encounters.

From an institutional perspective, communication strategies deliberately integrated digital and direct outreach.

“I distribute information through official websites and email, and it is reinforced through direct community meetings.”

(Health communication officer, 30 years, Sumenep)

Similarly, digital dissemination was viewed as most effective when accompanied by direct engagement.

“The most effective way is still direct education in front of the community, but supported by digital media so the information can reach more people.”

(Local social media actor, 26 years old, Sumenep)

At the community level, mothers and local figures described receiving information through both channels.

“Usually I get nutrition information from Instagram or WhatsApp groups, and also from training sessions and monthly Posyandu meetings.”

(Female community figure, 26 years old, Sumenep)

“I learned from pre-marital classes at the health centre, but I also searched on Google and Instagram.”

(Bride-to-be, 19 years, Sumenep)

Religious leaders similarly combined offline gatherings with simple digital tools.

“I usually share nutrition information through religious gatherings and WhatsApp groups at the pesantren.”

(Religious leader, 52 years, Sumenep)

Health cadres emphasised that digital coordination complemented, rather than substituted for, face-to-face education.

“Sometimes I get information from the Posyandu WhatsApp group, but most education happens during face-to-face meetings.”

(Health cadre, 42 years, Sumenep)

“We share information directly during Posyandu activities, home visits, women’s gatherings, and religious meetings, and sometimes through WhatsApp groups when information needs to be spread quickly.”

(Health cadre, 31, Pamekasan)

Public health officers also described this integration of channels.

“I share information through WhatsApp groups with health cadres, and education is delivered through mobile Posyandu activities.”

(Public health officer, 34 years, Sumenep)

Village midwives reinforced this pattern of dual communication.

“I share nutrition information through direct counselling at Posyandu, home visits, and during antenatal or immunisation check-ups. I also communicate with health cadres and mothers’ groups through WhatsApp to reinforce key messages.”

(Midwife, 40, Pamekasan)

Participants further highlighted the importance of offline validation when encountering online information.

“If I feel confused, I usually ask the midwife or Posyandu cadre directly. Online information helps, but a direct explanation is still important.”

(Community leader, 52, Pamekasan)

Across accounts, face-to-face meetings at Posyandus, home visits, religious gatherings, and community forums laid the foundation for trust. Digital platforms such as WhatsApp, Instagram, and messaging groups were used to reinforce reminders, circulate updates, and maintain ongoing contact between formal health actors and families.

Gender-sensitive serious games were perceived as most acceptable when embedded in existing routines rather than introduced as standalone interventions. This hybrid model enabled the circulation of nutrition messages across multiple communication spaces while remaining anchored in trusted interpersonal relationships. (Informants: Mothers, cadres, health workers, religious leaders, and digital communicators, ages 26–53, across Bangkalan, Sumenep, and Pamekasan)

### 3.3. Perceptions of Digital Health Technologies and AI-Supported Communication

Participants expressed nuanced and conditional views toward digital health technologies, including AI-supported communication tools. Across participant groups, digital technologies were not perceived as standalone solutions, but as tools whose usefulness depended on trust, cultural relevance, accessibility, and alignment with existing community-based health communication structures.

#### 3.3.1. AI as a Supportive Tool Rather than a Replacement for Health Workers

Across informant groups, AI-based tools were consistently perceived as supportive innovations rather than substitutes for human health workers. Participants highlighted that while AI could enhance access to information, reminders, and efficiency, interpersonal interaction and contextual understanding remained essential.

Mothers highlighted the importance of direct explanation and dialogue.

“I don’t think AI can fully replace health workers, because people still need direct explanations and opportunities to ask questions.”

(Female community figure, 26 years old, Sumenep)

Similarly, a mother of an under-five child stated:

“AI can help provide information, but not replace health workers in all aspects.”

(Mother of under-five children, 40 years, Sumenep)

Younger participants expressed openness to AI-based tools while emphasising the need for professional oversight.

“AI can give information quickly, but it cannot fully replace doctors.”

(Bride-to-be, 19 years, Sumenep)

Some participants viewed AI as capable of delivering information, but not performing clinical or contextual roles.

“AI can replace health workers in providing information, but examinations and other health services still require medical professionals.”

(Local social media actor, 26 years old, Sumenep)

Health cadres framed AI as a complementary aid in community-level education.

“AI cannot replace medical personnel, but it can help cadres provide information.”

(Health cadre, 42 years, Sumenep)

“AI can be a very good supporting tool, but it cannot fully replace health workers. Machines cannot replace personal interaction, empathy, and understanding local social conditions.”

(Health cadre, 31, Pamekasan)

Village midwives reinforced this perspective by emphasising the irreplaceability of empathy and contextual judgement.

“AI cannot fully replace health workers. It can be a powerful supporting tool by providing data and initial recommendations, but empathy, personal interaction, and understanding family contexts are things that only health workers can provide.”

(Midwife, 40, Pamekasan)

Institutional actors similarly framed AI as an efficiency-enhancing layer rather than an autonomous decision-maker.

“AI cannot replace doctors or midwives; it only supports their work.”

(Health communication officer, 30 years, Sumenep)

Religious leaders extended this logic beyond clinical authority to moral authority.

“AI can be used to help children’s health, but it cannot replace religious leaders.”

(Religious leader, 52 years, Sumenep)

Collectively, these accounts illustrate a consistent expectation that AI-based systems should function as assistive tools integrated within existing trust-based care relationships. Technology was accepted as useful for information retrieval, monitoring, and reminders, but final guidance, reassurance, and interpretation were expected to remain with trusted human actors. Trust, empathy, and contextual negotiation were repeatedly described as relational qualities that technology alone could not provide. (Informants: Mothers, brides-to-be, cadres, midwives, religious leaders, and digital communicators, ages 19–53, across Bangkalan, Sumenep, and Pamekasan)

#### 3.3.2. Trust, Accuracy, and Cultural Fit as Conditions for Acceptance

Across informants, acceptance of AI-supported nutrition tools was consistently described as conditional rather than automatic. Trust depended on perceived accuracy, verification by health professionals, institutional endorsement, and alignment with local cultural and religious values.

Institutional actors emphasised that unverified technological dependence could be problematic.

“The main concern is excessive dependence on technology without verification by health workers.”

(Health communication officer, 30 years, Sumenep)

Similarly, a midwife highlighted broader concerns about misinformation circulating in digital spaces:

“There is a lot of inaccurate and misleading nutrition information on social media. I always advise the community not to believe it immediately and to verify it with health professionals.”

(Midwife, 40, Pamekasan)

For many participants, credibility was closely linked to medical endorsement and official sources.

“AI should be trustworthy, sourced from health professionals, and balanced with information from medical staff.”

(Local social media actor, 26 years old, Sumenep)

“Sometimes the information on social media is confusing. If it comes from official government accounts or health organisations, then I trust it.”

(Community leader, 52, Pamekasan)

Mothers and brides-to-be described cautious but open attitudes toward AI-based tools, provided that explanations were clear and verifiable.

“If the explanation is easy to understand, I feel comfortable, but it still needs to be checked for accuracy.”

(Female community figure, 26 years old, Sumenep)

“I am interested in using AI because I could ask questions directly, but I am afraid of getting incorrect information.”

(Mother, 31, Sumenep)

“At the beginning, people may find it difficult to trust AI, but with proper education, they will accept it.”

(Mother of under-five children, 40 years, Sumenep)

“I worry that AI is not updated with the latest information. People will trust it if it is official.”

(Bride-to-be, 19 years, Sumenep)

Several participants explicitly stated that digital content was often cross-checked with trusted local actors before being accepted.

“There is a lot of nutrition information on the internet, but I am afraid it might be incorrect. Usually, I wait until the cadre or midwife explains it.”

(Mother A, 24)

“Not everyone will trust digital applications.”

(Mother SK, 29)

“I sometimes see health information on social media, but I am not sure whether it is correct.”

(Bride-to-be, 20)

Cultural and contextual alignment was repeatedly identified as a prerequisite for trust. Participants were sceptical of generalised recommendations that did not reflect local food practices.

“People will not trust AI if there is no explanation, especially if it is not adapted to local conditions.”

(Health cadre, 42 years, Sumenep)

“The main concern is the accuracy of the information—whether it matches local conditions and eating cultures in our village.”

(Health cadre, 31, Pamekasan)

Religious leaders further emphasised the importance of alignment with moral and spiritual frameworks.

“I worry that AI might spread information that is not in line with religious teachings.”

(Religious leader, 52 years, Sumenep)

Taken together, these accounts suggest that trust in AI-supported nutrition tools is socially mediated and culturally negotiated. Acceptance depends not only on technical functionality or perceived usefulness, but also on institutional legitimacy, contextual relevance, and endorsement from trusted health and religious actors. Digital tools perceived as generic, culturally insensitive, or lacking verification were approached with caution. Conversely, AI systems integrated within existing community-based health structures were viewed as more credible and potentially acceptable. (Informants: Mothers, brides-to-be, cadres, midwives, religious leaders, and digital communicators, ages 19–52, across study sites)

#### 3.3.3. Digital Literacy and Infrastructure Constraints

Practical limitations related to digital literacy and infrastructure were also highlighted. Shared device use and unstable internet connections influenced how participants engaged with digital health resources.

“The phone is shared in our household, and sometimes the signal is poor. That is why it is easier to ask directly.”

(Mother, A, 24)

“People need time to understand that now everything is technology-based.”

(Mother SK, 29)

Digital adoption remained uneven, with infrastructural limitations and resistance to change shaping community responses to new technologies.

“It is difficult, but it can be attempted; however, facilities and people’s readiness are still major obstacles.”

(Local social media actor, 26 years old, Sumenep)

Participants also identified practical barriers that shaped engagement with digital health technologies. Limited access to stable internet connections, shared use of mobile devices within households, and varying levels of digital literacy, particularly among older caregivers, were cited as challenges. These constraints influenced how frequently and effectively digital tools could be used in everyday contexts.

Differences in digital readiness across community groups were identified as key challenges.

“Some people are ready to use technology, especially younger mothers, but others still need assistance because of low digital literacy.”

(Midwife, 40, Pamekasan)

These constraints shape how digital tools can be realistically implemented in rural contexts.

Limited familiarity with advanced digital technologies shaped expectations and usage patterns.

“I do not really understand advanced technology like AI. I only use simple applications such as WhatsApp or watching videos on YouTube.”

(Community leader, 52, Pamekasan)

This reflects how digital literacy influences engagement with health technologies.

Limited digital literacy and uneven internet access were perceived as key barriers to the wider adoption of AI-supported health communication.

“Many people are still not familiar with technology, so it needs to be introduced slowly through cadres and community meetings.”

(Female community figure, 26 years old, Sumenep)

Digital literacy and uneven readiness across age groups were identified as challenges.

“Younger generations may be more ready, but older people or those who are not familiar with smartphones need special assistance.”

(Health cadre, 31, Pamekasan)

Healthcare professionals observed that while younger participants were generally more comfortable navigating digital platforms, older family members often required assistance, underscoring the importance of simple interfaces and offline or low-data features.

Digital literacy and uneven internet access emerged as major constraints, particularly among older adults and rural households. While younger participants demonstrated greater confidence in using digital tools, others required guidance. These disparities highlighted the importance of blended approaches and ongoing support. (Informant 1—Mother, 42 years, Bangkalan; Informant 5—Community leader, 51 years, Bangkalan)

Limited digital literacy and unequal access to technology were identified as barriers, particularly among older community members. These constraints influenced who could benefit from digital nutrition communication. (Ummi, mother of an under-five child, 29 years, Bangkalan; Usman Yunus, bride-to-be, 19 years, Bangkalan)

Limited digital literacy and inconsistent internet access were identified as key barriers, particularly among older adults and rural residents. While younger participants demonstrated higher confidence in navigating digital platforms, others required guidance and repeated exposure to build familiarity and trust. (Informants: Community members and cadres, ages 26–53, rural Madura)

#### 3.3.4. Expectations for Human Mediation in Digital Health Tools

Participants consistently emphasised that AI-based tools should not operate independently, but rather be mediated, adapted, and introduced through trusted human actors. Expectations centred on simplicity of language, cultural sensitivity, consultation features, and integration within existing community health relationships.

Several informants highlighted the importance of accessible and youth-friendly communication styles.

“AI should use simple, youth-friendly language so it is easy to understand.”

(Bride-to-be, 19 years, Sumenep)

Similarly, clarity and contextual adaptation were emphasised:

“AI would be better if it worked together with the midwife. It should use simple language so everyone can understand.”

(Mother, 31, Sumenep)

Beyond language, participants expected AI tools to be mediated through trusted health professionals and community actors rather than delivered as standalone technologies.

“Medical professionals should deliver AI in ways that align with the local community environment.”

(Local social media actor, 26 years old, Sumenep)

“Technology should involve health cadres and community leaders so that people feel guided and confident when using it.”

(Midwife, 40, Pamekasan)

Participants also stressed the need for built-in consultation features that allow direct interaction with experts.

“There should be a consultation feature, and intensive socialisation is needed so people can accept AI.”

(Health cadre, 42 years, Sumenep)

“There should be a direct chat feature with experts so the application is more trusted.”

(Mother of under-five children, 40 years, Sumenep)

A gradual introduction through trusted intermediaries was viewed as essential to acceptance.

“AI-based technology should be introduced gradually and through cadres or midwives who are already trusted by the community.”

(Health cadre, 31, Pamekasan)

Religious alignment was also identified as an important consideration, particularly when discussing educational or game-based formats.

“Educational games must be designed in a way that respects religious values.”

(Religious leader, 52 years, Sumenep)

Across participant groups, digital technologies were framed as complementary tools embedded within existing social systems. Rather than engaging independently with AI-based applications, participants expressed greater confidence when digital tools were introduced, explained, or endorsed by health cadres, village midwives, or community leaders. Human oversight was perceived as necessary to build trust, prevent misinterpretation, and ensure that nutrition guidance remained contextually appropriate. (Informants: Mothers, brides-to-be, cadres, midwives, religious leaders, and digital communicators, ages 19–53, across study sites)

### 3.4. Perceived Opportunities for Gender-Sensitive Serious Games in Nutrition Communication

Participants articulated a range of perceived opportunities for using serious games in nutrition communication in rural contexts. Importantly, these perspectives did not frame serious games as standalone interventions or replacements for existing health services. Instead, participants described them as potential complementary tools that could support engagement, reinforce messages, and align nutrition communication with everyday social and cultural practices.

#### 3.4.1. Serious Games as Complementary Communication Tools

Serious games were consistently described as complementary rather than substitutive tools within existing nutrition communication systems. Informants indicated that digital games would be acceptable only if integrated into established structures, such as Posyandu sessions, home visits by health cadres, and consultations with village midwives.

“If all approaches are combined, it would be better, but there still needs to be consultation with the midwife.”

(Mother, 31, Sumenep)

This reflects a relational understanding of nutrition communication in which authority remains embedded in trusted human actors. Serious games were not expected to function independently but to operate alongside interpersonal guidance, serving as reinforcement or reminders rather than primary sources of legitimacy.

In addition to their complementary role, participants viewed educational games as engaging entry points for introducing nutrition concepts, particularly among younger audiences.

“Educational games make learning fun and not boring.”

(Bride-to-be, 19 years, Sumenep)

“Educational games are very good because children can learn while playing, so they don’t get bored, and the message is easier to absorb.”

(Female community figure, 26 years old, Sumenep)

Participants perceived serious games as potentially valuable tools for increasing attention and facilitating shared learning between mothers and children when embedded within broader educational efforts. (Informants: Mothers, brides-to-be, cadres, and media administrators, ages 19–40)

#### 3.4.2. Gamification and Nudging for Sustained Engagement

Participants discussed gamification elements such as points, rewards, reminders, and small incentives as features that could increase enthusiasm and sustained engagement with nutrition-related content. These mechanisms were perceived as making information more interactive and motivating within everyday routines.

“I am interested in applications with points or rewards because they help make nutrition monitoring more controlled.”

(Local social media actor, 26 years old, Sumenep)

“I like applications with points. Daily challenges make me more enthusiastic.”

(Bride-to-be, 19 years, Sumenep)

“If there is an application that gives points or rewards, I would be more enthusiastic.”

(Mother, 31, Sumenep)

Gamified features were also perceived as potentially reinforcing participation among community actors.

“Applications with points are good. Daily challenges and small reminders from cadres are very effective.”

(Health cadre, 42 years, Sumenep)

“If there are points or rewards for recording children’s nutrition, mothers will be much more motivated. Rewards make them feel that their efforts are appreciated.”

(Health cadre, 31, Pamekasan)

“If there are points or rewards, it can increase motivation among mothers and cadres.”

(Female community figure, 26 years old, Sumenep)

From a programmatic and institutional perspective, nudging strategies were described as practical tools for maintaining attention.

“Small reminders, such as SMS messages, have proven to be helpful.”

(Health communication officer, 30 years, Sumenep)

“Visual reminders like posters or stickers really help. If there are rewards or points, people will be even more motivated.”

(Community leader, 52, Pamekasan)

Religious framing was also perceived as a motivational dimension.

“Small daily reminders from religious teachers can be very effective.”

(Religious leader, 52 years, Sumenep)

Material incentives were mentioned as additional motivating factors in community settings.

“Incentives such as food packages or shopping vouchers can strongly motivate mothers to attend Posyandu and apply healthy nutrition practices.”

(Midwife, 40, Pamekasan)

Across participant groups, gamification and nudging were framed not as guarantees of behavioural outcomes, but as mechanisms that could support attention, reinforce routines, and make nutrition-related messages more approachable. Points, reminders, symbolic rewards, and small incentives were perceived as ways to sustain engagement without replacing interpersonal guidance. (Informants: Mothers, brides-to-be, cadres, midwives, community leaders, and digital communicators, ages 19–52)

#### 3.4.3. Intergenerational and Family-Based Learning Dynamics

Participants highlighted that nutrition learning within rural households often involves multiple generations. Differences in digital readiness were observed across age groups.

“Young people are ready for technology, but older people may not be.”

(Bride-to-be, 19 years, Sumenep)

Despite generational differences, participants emphasised that children could play an active role in learning processes when engaged through interactive formats.

“Children can be involved in nutrition games, but the impact may remain limited to knowledge unless families support it.”

(Local social media actor, 26 years old, Sumenep)

Educational games were widely perceived as appropriate entry points for engaging children.

“Games for children are good, so they like vegetables and can learn nutrition while playing.”

(Mother, 31, Sumenep)

“Children prefer learning through games. When learning is fun, they do not feel forced.”

(Community leader, 52, Pamekasan)

“Educational games can help children learn healthy eating habits in a fun way and encourage them to adopt these habits from an early age.”

(Midwife, 40, Pamekasan)

“Educational games are a brilliant idea. Children learn most effectively through play, and games can encourage them to like vegetables or fruits.”

(Health cadre, 31, Pamekasan)

Faith-based narratives were also perceived as culturally appropriate for family engagement.

“Children can be involved through Islamic nutrition stories.”

(Religious leader, 52 years, Sumenep)

Participants consistently framed children not merely as recipients of information but as active co-learners within family contexts. Game-based formats were described as facilitating curiosity, attention, and shared interaction between caregivers and children. Rather than implying direct behavioural outcomes, participants described engagement, discussion, and shared understanding within everyday routines.

Family involvement was repeatedly described as central to sustaining nutrition-related practices.

“If the family supports it, mothers are more enthusiastic about applying healthy eating practices.”

(Female community figure, 26 years old, Sumenep)

“Children also need to be involved, and small changes made consistently can have a big impact.”

(Health cadre, 42 years, Sumenep)

“Children can be involved from an early age, and small changes can be very meaningful if done consistently.”

(Public health officer, 34 years, Sumenep)

“The role of families and cadres is very important in assisting nutrition practices.”

(Health communication officer, 30 years, Sumenep)

These accounts illustrate that nutrition communication was understood as relational and family-based rather than individual. Participants described learning as occurring through shared activities, dialogue, and routine interactions among parents, children, and, at times, elders.

Overall, serious games were perceived as potentially supportive tools for facilitating intergenerational engagement. Rather than functioning as standalone educational mechanisms, they were viewed as media that could stimulate shared attention, reduce resistance, and create opportunities for family-based learning within trusted social environments. (Informants: Mothers, brides-to-be, cadres, midwives, religious leaders, and community figures, ages 19–52)

**Figure 3:**
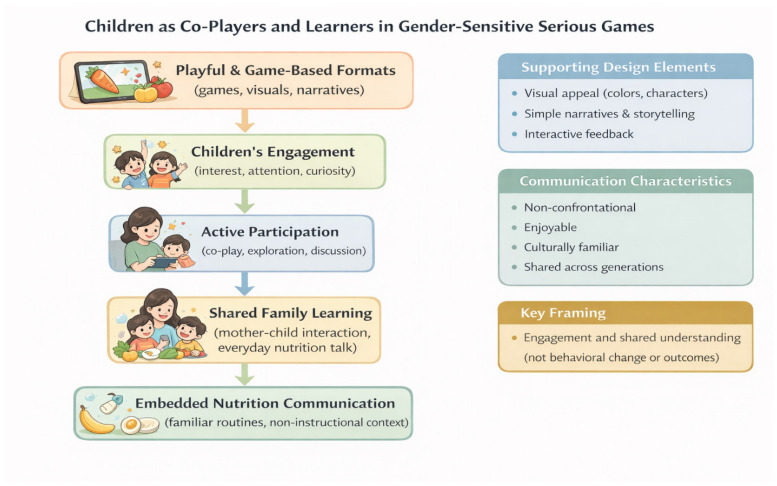
Conceptual representation of intergenerational nutrition learning dynamics in rural Madura.

These intergenerational learning dynamics are conceptually illustrated in Figure 3.

Participants described children as enthusiastic users of game-based formats and noted that interactive and playful approaches were more engaging for children than conventional information delivery. Mothers observed that children tended to show interest and attentiveness when nutrition-related messages were presented through games or playful activities, particularly when these activities involved shared participation with caregivers. In this context, participants perceived serious games as potentially relevant platforms for engaging children alongside mothers, creating opportunities for shared learning experiences within households. Rather than positioning children solely as recipients of instruction, game-based formats were viewed as enabling active participation, curiosity, and discussion around food choices and daily routines. Participants also noted that involving children in playful learning activities could support mothers’ efforts to introduce nutrition-related messages in everyday contexts. These perspectives highlight the importance of treating children as co-players and learners when designing gender-sensitive serious games for nutrition communication, without implying changes in behaviour or outcomes. Instead, the emphasis remains on engagement, shared understanding, and integrating learning into familiar family interactions.

#### 3.4.4. Cultural and Religious Adaptation in Game Narratives

Participants consistently emphasised that cultural sensitivity and alignment with religious beliefs were prerequisites for the acceptability of serious games in rural settings. Digital content was not perceived as neutral; rather, it was expected to reflect local values, moral norms, and familiar communication styles.

“Messages must be simple and involve religious leaders and cadres so the community accepts them.”

(Health cadre, 42 years, Sumenep)

“Educational games must be designed in a way that respects religious values.”

(Religious leader, 52 years, Sumenep)

Religious norms were described as central reference points in evaluating new communication tools. Participants indicated that content perceived as misaligned with religious teachings could face resistance, regardless of its educational purpose.

“Religious and community guidance is important so people trust the program.”

(Bride-to-be, 19 years, Sumenep)

Language and symbolic representation were also considered critical elements of cultural adaptation. Informants noted that localised expressions and familiar cultural cues could enhance relatability and acceptance.

“Simple messages using the Madurese language are more attractive to the community.”

(Public health officer, 34 years, Sumenep)

Beyond content design, participants highlighted the importance of culturally familiar delivery spaces. Community-based forums such as Posyandu, religious gatherings, and women’s groups were considered appropriate settings for introducing or reinforcing nutrition-related messages.

“Nutrition campaigns through mothers’ gatherings are effective because many women come and can discuss together.”

(Mother, 31 years, Sumenep)

“Meetings such as religious gatherings and women’s groups are very effective because people feel comfortable discussing and sharing experiences there.”

(Community leader, 52 years, Pamekasan)

“Community gatherings such as Posyandu, women’s groups, and religious meetings are effective because people feel comfortable and trust the information shared there.”

(Midwife, 40 years, Pamekasan)

“Community forums such as arisan, religious gatherings, and PKK meetings are very effective because people trust each other there.”

(Health cadre, 31 years, Pamekasan)

Institutional collaboration was also deemed necessary to ensure a smooth implementation and legitimacy.

“Religious leaders, cadres, midwives, and village heads must be involved so programs can run smoothly.”

(Health communication officer, 30 years, Sumenep)

Collectively, these accounts indicate that serious games must be culturally grounded and religiously sensitive to function as supportive communication tools. Rather than introducing externally framed or generic narratives, participants highlighted alignment with local language, moral values, and established community structures. Cultural and religious adaptation was therefore perceived not as an optional feature, but as a central condition for acceptability in conservative rural contexts.

Overall, participants’ accounts suggest that gender-sensitive serious games are perceived not as interventions in themselves, but as potential complementary tools embedded within trust-based, community-centred communication systems. These perceived opportunities provide a formative basis for discussing design considerations and theoretical implications, which are elaborated in the Discussion section that follows.

**Figure 4:**
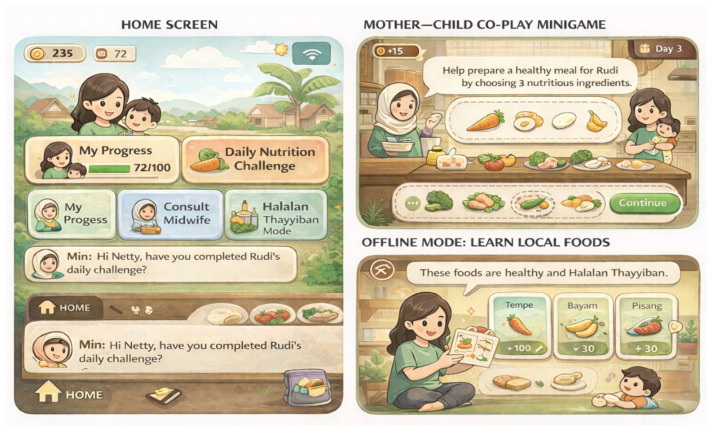
Conceptual mock-up visualising potential interface elements of the gender-sensitive serious game, developed based on formative qualitative findings. This mock-up does not represent a tested intervention, but a conceptual synthesis grounded in participants’ narratives and thematic findings.

Taken together, these thematic findings illustrate how gender-sensitive serious games may function not as standalone technological interventions, but as culturally embedded, family-oriented, and complementary communication tools. To synthesise these design imperatives into a visual representation, a conceptual interface mock-up was developed based on participants’ narratives and contextual insights (see Figure 4).

## 4. Discussion

### 4.1. Trust-Based Nutrition Communication and Gendered Caregiving Contexts

This study highlights that nutrition communication in rural Madura is deeply embedded in trust-based and gendered caregiving structures. Mothers were consistently identified as the primary actors responsible for daily food preparation, child feeding, and health-related decisions, even when broader household authority involved spouses or elders. This positioning places mothers at the intersection of responsibility, accountability, and social expectation, shaping how nutrition information is interpreted and acted upon.

Rather than framing mothers as passive recipients of information, the findings emphasise their active role as negotiators of multiple, sometimes conflicting, sources of guidance, including family traditions, social media, and formal health advice. Consistent with previous studies in low- and middle-income settings, gendered caregiving responsibilities often coincide with emotional and cognitive burdens, particularly when nutrition outcomes are publicly evaluated [19,20]. Broader research on gendered parenting patterns also indicates that differentiated maternal and paternal roles shape family-level decision-making and youth-related outcomes across contexts [19].

Within this context, participants perceived serious games not as solutions to nutritional challenges, but as potentially supportive communication tools that could complement mothers’ existing caregiving roles. Importantly, participants emphasised the need for approaches that acknowledge structural constraints and shared responsibilities, and that avoid narratives that place sole blame on mothers for child nutrition outcomes. These findings suggest that trust-sensitive and gender-aware framing is central to the conceptualisation of digital nutrition communication in rural settings.

### 4.2. Community and Institutional Mediation as Foundations of Credibility

Findings from this study underscore that trust in nutrition communication is not generated solely through digital platforms, but through social and institutional mediation. Health cadres, village midwives, religious leaders, and community figures were consistently described as trusted intermediaries who legitimise nutrition-related messages within rural Madura. Participants emphasised that digital tools, including serious games, were perceived as more acceptable when visibly associated with trusted institutions such as puskesmas or introduced through familiar community actors, such as cadres or ustadzah. Rather than functioning independently, digital communication tools were viewed as extensions of existing health communication ecosystems.

This perspective aligns with research indicating that institutional endorsement and interpersonal relationships play a critical role in shaping trust toward digital health initiatives. In this study, trust emerged as a socially produced process rather than a technological feature. These findings caution against standalone digital interventions and highlight the importance of embedding communication innovations within established community and institutional networks [20].

These findings resonate with community health mediation frameworks, which conceptualise community health workers and local intermediaries as relational brokers who translate biomedical knowledge into culturally meaningful practices within marginalised or rural populations [20]. Rather than functioning merely as information distributors, mediators serve as trust builders, cultural interpreters, and facilitators of dialogue between formal health systems and community-based social structures.

### 4.3. AI-Supported Features Within Gender-Sensitive Serious Games

Participants’ perceptions of AI-supported features within gender-sensitive serious games positioned these elements as supportive rather than autonomous.

Across informant groups, AI and digital applications were described as helpful for providing reminders, organising information, or supporting awareness, but not as replacements for human judgement, cultural understanding, or interpersonal interaction.

Concerns regarding accuracy, cultural relevance, and digital literacy were frequently raised, particularly regarding limitations in rural infrastructure and uneven access to technology. Participants emphasised that usability, simplicity, and offline compatibility were essential conditions for acceptance. Importantly, digital literacy constraints were framed not as user deficits but as contextual factors that should inform design choices.

These findings reinforce the existing literature cautioning against technocentric assumptions in digital health. In rural Madura, participants perceived the legitimacy of digital tools as contingent upon human mediation, institutional validation, and alignment with local realities. This reinforces the interpretation of AI and digital platforms as assistive communication layers rather than independent agents of change.

Similar patterns have been observed in rural health contexts, where AI-based systems enhance efficiency and information access but remain dependent on human mediation and local infrastructure [21].

### 4.4. Serious Games as Family-Centred and Gender-Sensitive Communication Spaces

Participants described serious games as potentially meaningful communication spaces when conceptualised as family-centred, interactive, and non-instructional. Shared gameplay between mothers and children was perceived as an enjoyable context for encountering nutrition-related messages without reinforcing hierarchical or didactic communication patterns. Rather than positioning children as passive recipients of instruction, participants viewed them as active co-players whose curiosity and engagement could support shared learning within households. Mother–child co-play was perceived as easing emotional strain on caregivers and reducing resistance often encountered in direct guidance. These perceptions align with studies suggesting that collaborative and playful learning environments may enhance engagement and attention [22].

Previous research in low- and middle-income settings has demonstrated that culturally adapted serious games can improve maternal health knowledge among low-literacy populations. For instance, the MANTRA project in rural Nepal reported gains in knowledge of maternal and neonatal health through co-created mobile game modules. Meanwhile, similar initiatives in rural Ethiopia highlighted the potential of serious games to support maternal healthcare education in resource-constrained contexts. These studies suggest the feasibility of serious games in comparable settings.

Importantly, however, participants in the present study did not frame serious games as standalone interventions or direct behaviour-change tools. Instead, games were conceptualised as relational communication spaces embedded within trust-based household and community shared understanding within everyday family routines, rather than producing measurable behavioural outcomes. This framing aligns with the exploratory and formative nature of the present study and avoids intervention-oriented claims.

### 4.5. Brides-to-Be and Young Women as Emerging Communication Audiences

Brides-to-be and young women were identified as a distinct and important audience for nutrition communication, given early marriage practices and limited preparedness for caregiving roles. Participants described this life stage as characterised by heightened vulnerability, limited autonomy, and reliance on senior family members for guidance.

Within this context, serious games were perceived as potentially relevant pre-parenting communication tools that allow private, low-pressure engagement with nutrition-related information. Rather than framing games as empowerment interventions, participants discussed their value as neutral reference points that could support confidence and dialogue without directly challenging household hierarchies.

Participants also noted higher digital familiarity among younger women, which shaped openness to game-based formats. However, digital familiarity was interpreted as a contextual opportunity rather than a guarantee of learning or change. These findings emphasise the importance of designing communication tools that are non-judgmental, exploratory, and culturally appropriate for young women navigating complex social expectations.

### 4.6. Design Implications for Gender-Sensitive Serious Games

Synthesising participants’ perspectives, several design considerations emerge for conceptualising gender-sensitive serious games for nutrition communication. These include localised storylines reflecting rural daily life, visual and gamified cues that support accessibility, modest and culturally relevant rewards, and interfaces designed for simplicity and offline compatibility. Participants emphasised that design features should align with everyday routines, collective norms, and existing community practices rather than introducing unfamiliar or technologically demanding systems. Religious and cultural alignment—such as references to halalan thayyiban or moderation—was also perceived as enhancing legitimacy and acceptance when integrated subtly into narratives. These considerations do not constitute tested design solutions but rather formative insights grounded in qualitative data. Together, they inform a conceptual framework for future development without implying effectiveness or implementation outcomes.

### 4.7. Methodological and Practical Limitations

This study has several limitations that should be acknowledged. First, the findings are based on qualitative data and reflect participants’ perceptions and expectations rather than observed behaviours or evaluated outcomes. Second, no prototype of a serious game was developed, implemented, or tested during this research. As such, conclusions are limited to conceptual design considerations rather than usability or impact assessment. The study is also context-specific, focusing on rural communities in Madura, which may limit transferability to other settings. Additionally, although multiple stakeholder perspectives were included, power dynamics and social desirability may have influenced participants’ responses. Additionally, social desirability bias may have influenced participants’ openness to digital tools, particularly when discussing AI-based or government-related technologies in the presence of researchers.

In addition, digital infrastructure and uneven smartphone access in rural areas may constrain the practical feasibility of AI-supported or game-based nutrition tools. Infrastructure constraints and digital access disparities documented in rural AI implementation studies may also influence the feasibility of technology-based nutrition communication in similar contexts. These structural considerations should be taken into account in future prototype development and pilot testing phases.

Therefore, future prototype development should proceed through a phased, participatory co-design approach involving community actors, rather than through rapid, top-down technological implementation.

### 4.8. Implications for Future Research and Development

As a formative qualitative study, this research provides a foundation for subsequent phases of inquiry. The next step will involve developing prototypes and pilot testing serious game concepts derived from the design principles identified here. Future research should explore usability, acceptability, and contextual fit through participatory design approaches and small-scale pilots before considering broader implementation. Such phased development aligns with recommendations for responsible digital health innovation, emphasising equity, cultural sensitivity, and contextual adaptation. Rather than advancing claims of effectiveness, future studies should focus on understanding how gender-sensitive serious games are experienced, negotiated, and integrated into existing health communication practices. Future development should align with broader digital health governance principles and lessons from rural AI deployment, particularly regarding phased implementation, infrastructure readiness, and community-level trust-building

Overall, this study highlights that the development of gender-sensitive serious games extends beyond technological innovation alone. It involves rethinking health communication to be more participatory, context-aware, and aligned with local cultural and social realities. Participants’ perspectives indicate that digital communication tools are more meaningful when they resonate with the lived experiences of mothers, children, and brides-to-be. Rather than positioning serious games as solutions to nutritional problems, this study frames them as communication resources that can support engagement and understanding when situated within existing community structures. The involvement of trusted actors, such as health cadres, midwives, and religious figures, was perceived as necessary to ground within familiar systems of authority and trust. In this sense, gender-sensitive serious games are conceptualized as part of an evolving landscape of public health communication that bridges traditional, relationship-based communication with emerging digital formats. This reflection underscores the importance of ensuring that digital health innovation remains attentive to inclusion, cultural relevance, and community context, particularly in rural settings

## 5. Conclusions

In line with the research objectives, this study explored how gender-sensitive serious games may be conceptualised as participatory nutrition communication tools for mothers, children, and brides-to-be in rural Indonesia. Rather than developing or evaluating a digital intervention, this qualitative exploratory study examined how gender relations, cultural norms, and community-based communication practices shape perceptions of digital nutrition communication in rural Madura. The findings illustrate how nutrition communication practices are embedded within gendered caregiving roles, household authority structures, and socially constructed expectations regarding women’s responsibilities for child health. By situating serious games within trust-based community structures, this study provides empirically grounded insights into how these tools may be perceived, negotiated, and contextualised in rural settings.

### 5.1. Summary of Findings

The findings indicate that mothers occupy central roles in daily child nutrition practices. Yet, their decision-making space is often shaped by patriarchal norms, household hierarchies, and elders’ influence. Brides-to-be and young women, many entering marriage at an early age, were described as beginning parenthood with limited exposure to structured nutrition information and constrained autonomy in food-related decisions. Children were perceived not only as recipients of nutrition messages but also as active learners who respond positively to playful and participatory communication formats. Participants highlighted that acceptance of gender-sensitive serious games for nutrition communication depends on community roles, trust, cultural familiarity, ease of use, and visible endorsement by community actors such as health cadres, midwives, and religious leaders. Digital technologies and AI-supported tools were viewed as assistive rather than autonomous communication layers. Overall, the findings emphasise that digital nutrition communication must be embedded within local social structures and relational trust networks. These results highlight the importance of integrating gender-sensitive communication approaches that acknowledge women’s caregiving roles while also engaging male community actors as supportive agents in nutrition communication.

### 5.2. Theoretical Contributions

This study contributes to theory in three key ways. First, drawing on feminist communication perspectives, it demonstrates that nutrition communication must be understood within structural and relational gender dynamics rather than framed as the transfer of individual knowledge to women. Second, by engaging with serious games scholarship, the study conceptualises gamification and nudging as communicative mechanisms that embed health-related messages within everyday family interactions, rather than as tools for behavioural control. Third, using technology acceptance frameworks (TAM/UTAUT), the findings extend existing models by highlighting the roles of cultural legitimacy, institutional validation, and community mediation in shaping acceptance in rural contexts [23]. In the rural Madurese context, perceptions of usefulness and ease of use do not operate independently but are strongly shaped by social legitimacy, endorsement from trusted actors, and cultural alignment. This suggests that technology acceptance is relationally and contextually embedded rather than purely individual.

### 5.3. Practical Implications for Design

Participants’ perspectives suggest several formative design considerations:

1. Localised narratives reflecting rural life, including familiar household routines and community settings.
2. Visual gamified cues that enhance accessibility, particularly for users with limited literacy.
3. Mother–child co-play mechanisms that support shared learning rather than hierarchical instruction.
4. Modest and culturally relevant recognition systems, including digital acknowledgement and community-based appreciation.
5. Integration of religious and cultural values, such as halalan thayyiban, to enhance legitimacy.
6. Simplicity and offline compatibility, ensuring usability on low-end devices and in low-connectivity environments.

These considerations represent formative insights to guide prototype development rather than tested implementation strategies.

### 5.4. Policy Implications

From a policy perspective, the findings suggest that serious games should not be positioned as standalone solutions for stunting prevention. Instead, they may be conceptualised as complementary communication tools integrated into existing institutional frameworks, such as family planning and maternal-child health programmes. Participants emphasised the importance of collaboration with health professionals, community leaders, and religious institutions to ensure trust and contextual relevance. Any future policy integration should proceed cautiously, prioritising the evaluation of usability, acceptance, and contextual fit before broader scale-up.

### 5.5. Limitations and Future Research

This study is limited by its qualitative design and its context-specific focus on rural Madura, which may limit its transferability to other settings. No serious game prototype was developed, implemented, or tested; therefore, conclusions are limited to perceptions and design considerations. Future research should adopt a participatory prototype-development approach, followed by pilot testing, to assess usability, acceptability, and contextual adaptation. Subsequent studies may also explore ethical considerations related to AI personalisation, data protection, and the safeguarding of vulnerable groups, such as children and young brides. Comparative research across different regions would further clarify how cultural variation shapes the reception of digital nutrition communication tools.

### 5.6. Final Reflection

This study does not claim behavioural change, intervention effectiveness, or measurable health outcomes. Instead, it offers a formative, context-sensitive understanding of how gender-sensitive serious games may be conceptualised within trust-based rural communication systems. The findings underscore that digital innovation cannot be detached from social structure, cultural legitimacy, and institutional mediation. In rural Madura, technology was not perceived as a substitute for human interaction but as a supportive layer that gains legitimacy through community validation. By grounding digital design considerations in lived experiences and relational trust dynamics, this study provides a cautious yet constructive foundation for future prototype development and empirical exploration. The next phase of research will focus on participatory co-design and pilot evaluation to examine how these conceptual insights translate into practical communication tools.

## Figures and Tables

**Table 1:** Participant Characteristics.

Participant Group	Approx Age Range (Years)	Gender	Location	Number
Mother of children under five	24–42	female (*n* = 8)	Madura Island, East Java	8
Brides-to-be/young women and men	19–25	male (*n* = 4), female (*n* = 3)	Madura Island, East Java	7
Health cadres/Villages midwives	26–53	male (*n* = 1), female (*n* = 13)	Madura Island, East Java	14
Opinion (community) leaders/religious leaders	26–52	male (*n* = 3), female (*n* = 4)	Madura Island, East Java	7
Social media actors	23–30	male (*n* = 4) female (*n* = 2)	Madura Island, East Java	6
Total				42

## Data Availability

The original contributions presented in this study are included in the article. Further inquiries can be directed to the corresponding author.
